# Effect of a 12-Week Structured Exercise Program on Aerobic Capacity and Flexibility in Cardiac Physiologic Pacing Pacemaker Patients

**DOI:** 10.7759/cureus.111898

**Published:** 2026-07-01

**Authors:** Kiran S Dhaygude, Poovishnu T Devi, Rushikesh S Patil, Janhavee S Brahmadande

**Affiliations:** 1 Department of Cardiopulmonary Sciences, Krishna College of Physiotherapy, Krishna Vishwa Vidyapeeth, Deemed To Be University, Karad, IND

**Keywords:** aerobic capacity, cardiac rehabilitation, flexibility, pacemaker, physiological pacing, physiologic pacing, six-minute walk test

## Abstract

Background: Cardiac conduction disorders causing bradyarrhythmias often require permanent pacing. Although physiologic pacing better preserves ventricular activation than conventional right ventricular pacing, many patients continue to experience reduced exercise capacity, flexibility, and functional independence after implantation. Despite the recognized benefits of cardiac rehabilitation, evidence regarding structured exercise programs in patients with cardiac physiologic pacing remains limited.

Objective: To evaluate the effects of a 12-week structured aerobic and flexibility exercise program on functional exercise capacity (Six-Minute Walk Test (6MWT)) and upper- and lower-limb flexibility in clinically stable adults with cardiac physiologic pacing, including Left Bundle Branch Area Pacing (LBBAP) and His Bundle Pacing (HBP).

Methods: A quasi-experimental single-group pre-post study was conducted at Krishna College of Physiotherapy. Sixty-one clinically stable adults (52-74 years), at least three months after pacemaker implantation, were recruited by convenience sampling. Participants completed a 12-week exercise program comprising 40-50-minute sessions, 3-4 times weekly, integrating aerobic and flexibility training. Functional exercise capacity was assessed using the 6MWT, upper-limb flexibility using the Back Scratch Test, and lower-limb flexibility using the Sit-and-Reach Test. Data were analyzed using paired t-tests in IBM SPSS Statistics for Windows, Version 25 (Released 2017; IBM Corp., Armonk, New York), with p < 0.05 considered statistically significant.

Results: Participants had a mean age of 59.72 ± 4.54 years; 52 (85.2%) were male, and 9 (14.8%) were female. After 12 weeks, significant improvements were observed in all primary outcomes. The mean 6MWT distance increased from 336.93 ± 18.90 m to 344.00 ± 26.59 m (t = 2.674, p = 0.0096). Upper-limb flexibility improved from −20.22 ± 4.80 cm to −19.09 ± 5.14 cm (t = 3.373, p = 0.0013), while lower-limb flexibility improved from −21.37 ± 4.67 cm to −18.32 ± 9.30 cm (t = 2.966, p = 0.0043). No major adverse events, device malfunctions, or lead-related complications occurred during the intervention.

Conclusion: A 12-week structured aerobic and flexibility program was feasible and associated with significant improvements in functional exercise capacity and flexibility in patients with cardiac physiologic pacing. No major adverse events occurred during the intervention. These findings provide preliminary evidence supporting structured exercise rehabilitation in this population; however, larger randomized controlled trials with longer follow-up are required to confirm these findings before routine clinical implementation can be recommended.

## Introduction

Normal myocardial function relies on precisely timed electrical propagation through the heart’s specialized conduction pathways [1]. Disturbances in impulse generation or transmission produce arrhythmias manifesting as bradycardia, tachycardia, or irregular rhythms that can diminish functional capacity and, in severe cases, lead to heart failure or sudden death. Among these disorders, conduction system abnormalities involving the sinoatrial node, atrioventricular node, bundle of His, or Purkinje network are clinically important because they interfere with normal electrical propagation and may lead to symptomatic bradyarrhythmias [2]. Patients commonly experience dizziness, syncope, fatigue, reduced exercise tolerance, and, in severe cases, heart failure or sudden cardiac death. For many of these conditions, permanent pacemaker implantation remains the most effective treatment option.

The increasing prevalence of conduction disorders worldwide is largely attributed to population aging and the growing burden of cardiovascular disease [3]. Traditional right ventricular pacing has been widely used for decades to manage symptomatic bradycardia; however, long-term stimulation of the right ventricle may disrupt the heart's natural activation sequence, resulting in ventricular dyssynchrony and an increased likelihood of pacing-induced ventricular dysfunction [4]. To overcome these limitations, cardiac physiologic pacing techniques such as Left Bundle Branch Area Pacing (LBBAP) have been developed. These approaches stimulate the intrinsic conduction system and maintain a more physiologic pattern of ventricular activation, thereby preserving cardiac performance and mechanical synchrony [5].

Although pacing therapy effectively restores heart rhythm, many patients continue to experience limitations in physical functioning after implantation. Reduced exercise capacity remains a common concern despite correction of the underlying rhythm abnormality. Several mechanisms contribute to this limitation, including impaired chronotropic response during exercise, reduced cardiovascular reserve, autonomic imbalance, peripheral muscle deconditioning, and suboptimal hemodynamic adjustments that result in inadequate heart rate augmentation, altered atrioventricular timing, impaired ventricular filling, and reduced exercise-related cardiac output [6-8]. In addition, long-term pacing-related mechanical alterations may impair circulatory efficiency and oxygen delivery, further contributing to deconditioning, diminished aerobic performance, and reduced functional independence [9].

Musculoskeletal impairments represent another important challenge following pacemaker implantation. During the early recovery period, patients are often advised to limit arm movements on the side of implantation to protect the pacing leads. While these precautions are clinically necessary, prolonged movement restriction may contribute to shoulder stiffness, reduced flexibility, and altered movement patterns. Patients may develop kinesiophobia after permanent pacemaker implantation due to concerns regarding device integrity or lead displacement, which may promote protective movement behaviors and reduced physical activity during recovery [10]. Over a period of time, these factors may lead to soft tissue tightness, reduced range of motion, and difficulty performing activities such as reaching, dressing, and grooming [11, 12].

Exercise-based cardiac rehabilitation has demonstrated beneficial effects on cardiovascular function, exercise capacity, and musculoskeletal recovery following various cardiac interventions, including in patients with conventional pacemakers [13, 14]. Aerobic training improves cardiovascular efficiency, enhances peripheral oxygen utilization, and increases functional exercise tolerance, while flexibility and mobility exercises help restore joint movement, improve tissue extensibility, and reduce stiffness associated with prolonged inactivity. Although these rehabilitation strategies have been shown to be safe and effective in conventional pacemaker recipients, evidence specifically evaluating patients with cardiac physiologic pacing remains limited. Because physiologic pacing preserves native ventricular activation and more physiologic cardiac mechanics than conventional right ventricular pacing, rehabilitation outcomes observed in conventional pacemaker populations may not be directly applicable. This gap in the literature provides the rationale for the present study.

To evaluate functional improvements following rehabilitation, reliable and clinically applicable outcome measures are required. The Six-Minute Walk Test (6MWT) is widely used to evaluate submaximal functional exercise capacity and provides valuable information regarding a patient's ability to perform everyday physical activities [15]. Flexibility can be assessed using field-based measures such as the Sit-and-Reach Test for lower limb and lower back flexibility and the Back Scratch Test for upper extremity and shoulder mobility [16].

Although exercise-based cardiac rehabilitation has demonstrated benefits in patients undergoing conventional cardiac interventions and traditional right ventricular pacing, evidence regarding structured exercise programs specifically for patients with cardiac physiologic pacing remains limited. Consequently, it is still unclear whether the improvements in aerobic capacity and flexibility reported in conventional pacemaker populations can be directly extrapolated to patients with physiologic pacing, who exhibit different patterns of ventricular activation and cardiac mechanics. To address this gap, the present study was undertaken to evaluate the effects of a 12-week structured aerobic and flexibility exercise program in clinically stable adults with cardiac physiologic pacing, including LBBAP and His Bundle Pacing (HBP). The primary outcomes evaluated were functional exercise capacity, assessed using the 6MWT, and upper- and lower-limb flexibility, assessed using standardized flexibility tests. We hypothesized that participation in the 12-week structured exercise program would result in significant improvements in functional exercise capacity and upper- and lower-limb flexibility following the intervention.

## Materials and methods

Study design and setting

This quasi-experimental investigation employed a single-group pre-post design with convenience sampling at Krishna College of Physiotherapy. The study was conducted from November 6, 2025, to February 8, 2026. The regimen combined aerobic and flexibility exercises in 40-50 minute sessions, scheduled 3-4 times weekly.

Participants

Sixty-one clinically stable adults of all genders, aged 52-74 years, who had undergone cardiac physiologic pacing, including LBBAP and HBP, at least three months before enrollment and were receiving stable medical therapy, were included in the study.

Exclusion criteria included recent infection, surgical procedures within three months pre-implant, terminal illness with life expectancy under one year, psychiatric conditions, or baseline walking performance >85% of predicted maximum. Written informed consent was obtained. Institutional ethics approval was obtained from the Institutional Ethics Committee of Krishna Vishva Vidyapeeth (Deemed to be a University), Karad (Approval No.: 274/2025-2026), before the commencement of the study. Written informed consent was obtained from all participants prior to enrollment.

Outcome measures

Six-Minute Walk Test

The 6MWT is a practical field-based assessment used to evaluate the functional capacity of individuals with cardiovascular and respiratory disorders. During the test, participants are instructed to walk continuously along a designated flat surface for six minutes while covering the maximum distance possible at a self-selected pace. The total distance walked is recorded as the primary outcome.

Unlike maximal exercise tests, the 6MWT reflects the ability to perform sustained submaximal physical activity similar to that required during daily living. The test assesses the combined performance of multiple physiological systems, which includes cardiovascular, pulmonary, circulatory, and musculoskeletal components. Due to its simplicity, safety, and minimal equipment requirements, it is commonly used in cardiac rehabilitation programs and clinical practice [17, 18].

Sit-and-Reach Test

The sit-and-reach test is a widely accepted method for evaluating flexibility of the hamstring muscles and lower back region. During assessment, the participant sits with the knees fully extended and slowly reaches forward toward or beyond the toes while maintaining proper alignment. The maximum distance achieved is measured and recorded in centimeters.

The test is easy to administer, requires minimal equipment, and has been extensively used in rehabilitation, fitness, and clinical settings. Previous investigations have reported good reliability and acceptable validity for assessing posterior chain flexibility in both healthy and clinical populations [19, 20]. Multiple recent literature pieces have further supported its usefulness for measuring hamstring flexibility and its high consistency [21]. In individuals with pacemaker implantation, assessment of lower body flexibility is particularly relevant because physical inactivity and fear of movement may contribute to reduced mobility and muscular tightness.

Back Scratch Test

The Back Scratch Test is a functional assessment used to examine shoulder mobility and upper-extremity flexibility. During the procedure, one arm reaches over the shoulder while the opposite arm reaches upward from behind the back. The distance between the fingertips is measured to evaluate the level of shoulder flexibility and mobility. Assessment of upper limb flexibility is especially important in patients who have undergone permanent pacemaker implantation because temporary movement restrictions and protective postures can lead to shoulder stiffness and reduced range of motion. The test provides a quick and clinically relevant measure of functional shoulder movement and is useful for monitoring individuals' improvement during rehabilitation.

Earlier studies have reported reliability and validity for shoulder mobility assessments using standardized testing procedures. Consistent positioning and administration techniques contribute to accurate measurement and make the test suitable for evaluating rehabilitation outcomes in clinical populations [22].

Procedure

A detailed screening procedure was completed with respect to inclusion and exclusion criteria before enrollment. Demographic data were obtained from each participant prior to assessment. All participants underwent pre-assessment using the selected outcome measures, including the 6MWT, Sit-and-Reach Test, and Back Scratch Test.

All participants received adequate counseling regarding the intervention program before the initiation of treatment. The structured exercise program consisted of aerobic, resistance, and flexibility training conducted over a period of 12 weeks. Aerobic exercise primarily involved walking and was performed 3-5 days per week for 20 minutes as part of each 40-50-minute exercise session. Exercise intensity was prescribed using the Borg Rating of Perceived Exertion (RPE) scale and was progressively advanced every four weeks from light to moderate and subsequently to vigorous intensity according to each participant's clinical tolerance and perceived exertion. Resistance exercises were performed 2-3 days per week and integrated with the aerobic and flexibility components. Flexibility training included stretching exercises for the neck, shoulder, and calf muscles, with each stretch held for 30 seconds and repeated three times. The initial exercise sessions were directly supervised by a qualified physiotherapist to ensure correct technique, safety, and adherence to the prescribed protocol. Thereafter, participants continued the program as a home-based regimen with regular telephone follow-up to monitor adherence, reinforce compliance, and address any concerns.

Thereafter, participants continued the program as a home-based regimen with regular telephone follow-up to monitor adherence, reinforce compliance, address any concerns, and identify any exercise-related adverse events throughout the intervention period.

The structured exercise program was designed according to the Frequency, Intensity, Time, and Type (FITT) principle. The detailed exercise prescription and progression protocol are summarized in Table 1.

**Table 1:** Structured 12-week exercise program based on the Frequency, Intensity, Time, and Type (FITT) principle. Exercise intensity was prescribed using the Borg Rating of Perceived Exertion (RPE) scale and progressed from light to moderate and subsequently to vigorous intensity at four-week intervals according to participant tolerance. Flexibility exercises consisted of static stretching with a 30-second hold for three repetitions for each muscle group. FITT: Frequency, Intensity, Time, and Type; RPE: Rating of Perceived Exertion.

FITT Principle	Phase I (Weeks 1-4)	Phase II (Weeks 5-8)	Phase III (Weeks 9-12)
Frequency	Aerobic exercise: 3-5 days/week Resistance exercise: 2-3 days/week	Same as Phase I	Same as Phase I
Intensity	Light intensity (Borg RPE 9-11)	Moderate intensity (Borg RPE 12-13)	Vigorous intensity (Borg RPE 14-16)
Time	Warm-up: 5 min; aerobic exercise: 20-30 min; resistance exercise: 15 min; static stretching: 30-second hold × three repetitions per muscle group; cool down: 5 min	Same as Phase I	Same as Phase I
Type	Walking, resistance exercises, and stretching exercises (neck, shoulder, and calf muscles)	Same as Phase I	Same as Phase I

Following completion of the 12-week intervention period, all enrolled participants (n = 61) successfully completed the study and underwent post-intervention assessment using the same outcome measures. No participant dropouts or missing outcome data were recorded; therefore, all participants were included in the final statistical analysis. The obtained data were subjected to statistical analysis, and pre-test and post-test differences were calculated to determine the impact of the intervention program.

Statistical analysis

Data analysis was completed using IBM SPSS Statistics for Windows, Version 25 (Released 2017; IBM Corp., Armonk, New York). Demographic characteristics were summarized using mean ± standard deviation for continuous variables and frequencies with percentages for categorical variables.

The comparison between pre-test and post-test outcome measures was performed with a paired t-test. As there were no participant dropouts or missing data, complete-case analysis was performed for all enrolled participants. Arithmetic mean and standard deviation were calculated for all outcome measures. A p-value less than 0.05 was considered statistically significant.

MS Excel (Microsoft Corporation, Redmond, Washington) was utilized for the preparation of graphical representations of frequencies and percentages.

## Results

Table 2 presents the demographic characteristics of the 61 participants included in the study. The mean age of the participants was 59.72 ± 4.54 years, indicating that the study population primarily consisted of middle-aged to older adults. The age distribution showed relatively low variability around the mean. With respect to gender, 52 participants (85.2%) were male, whereas nine participants (14.8%) were female. This demonstrates a marked predominance of male participants in the study sample. Overall, the study population was predominantly composed of older adult males, as presented in Table 2.

**Table 2:** Demographic characteristics of participants (n = 61).

Variables	Values (mean ± SD)/frequency
Age (years)	59.72 ± 4.54
Male	52 (85.2%)
Female	9 (14.8%)

Table 3 presents the baseline descriptive statistics of the study participants (n = 61). The mean 6MWT distance was 336.93 ± 18.90 m. The mean upper-limb and lower-limb flexibility scores were −20.22 ± 4.80 cm and −21.37 ± 4.67 cm, respectively.

**Table 3:** Descriptive statistics of overall participants (n = 61). 6MWT: Six-Minute Walk Test; UL: upper limb; LL: lower limb.

Variables	Values (mean ± SD)
6MWT	336.93 ± 18.90
Flexibility (UL)	−20.22 ± 4.80
Flexibility (LL)	−21.37 ± 4.67

Table 4 presents the comparison of pre-test and post-test outcome measures following the 12-week structured exercise program in patients with cardiac physiologic pacing pacemakers. The mean 6MWT distance increased from 336.93 ± 18.90 m to 344.00 ± 26.59 m. Upper limb flexibility improved from −20.22 ± 4.80 cm to −19.09 ± 5.14 cm, while lower limb flexibility improved from −21.37 ± 4.67 cm to −18.32 ± 9.30 cm. All outcome measures demonstrated statistically significant differences between pre-test and post-test assessments (all p < 0.05), as presented in Table 4.

**Table 4:** Comparison of pre-test and post-test data of the outcome measures. 6MWT: Six-Minute Walk Test; UL: upper limb; LL: lower limb.

Variables	Pre-test (mean ± SD)	Post-test (mean ± SD)	t-value	p-value
6MWT	336.93 ± 18.90	344 ± 26.59	2.674	0.0096
Flexibility (UL)	−20.22 ± 4.80	−19.09 ± 5.14	3.373	0.0013
Flexibility (LL)	−21.37 ± 4.67	−18.32 ± 9.30	2.966	0.0043

## Discussion

The current study evaluated the effects of a structured 12-week exercise program on functional exercise capacity and flexibility in patients who had undergone cardiac physiologic pacing pacemaker implantation. The results demonstrated significant improvements in aerobic performance as well as upper and lower limb flexibility following the intervention period. These findings suggest that appropriately prescribed exercise training can play a crucial role in enhancing physical function and reducing the impact of post-implantation inactivity.

The participants had a mean age of 59.72 ± 4.54 years, and most were male (85.25%). This demographic distribution reflects the higher occurrence of conduction system abnormalities and cardiovascular disorders in older adults, particularly among men [3, 15]. Although pacemaker implantation successfully corrects electrical conduction disturbances, restoration of normal rhythm alone does not always result in optimal functional recovery. Current management strategies increasingly focus on improving quality of life, physical activity levels, and independence, in addition to maintaining cardiac rhythm stability. A significant increase in 6MWT distance was observed after completion of the exercise program. The mean walking distance improved from 336.93 ± 18.90 m before training to 344.00 ± 26.59 m after training (t = 2.674, p = 0.0096). Reduced exercise tolerance is frequently reported in pacemaker recipients due to factors such as impaired chronotropic response, diminished cardiovascular reserve, skeletal muscle deconditioning, and fear of physical exertion [6, 11, 12]. Regular aerobic training promotes favorable cardiovascular and peripheral adaptations, including improved oxygen transport, enhanced muscle oxidative capacity, better endothelial function, and greater exercise efficiency [17, 18]. The observed improvement in walking performance suggests that a structured exercise program can be implemented safely in patients with cardiac physiologic pacing and may contribute to improved functional capacity, thereby supporting greater participation in daily activities and overall functional status [5, 6]. These findings are consistent with previous studies demonstrating that structured cardiac rehabilitation improves functional exercise capacity and walking performance in patients with cardiac implantable electronic devices [23]. Although the improvement in the 6MWT distance was statistically significant, the absolute increase was modest. Nevertheless, even small improvements in functional exercise capacity may contribute to enhanced mobility, greater independence in activities of daily living, and increased confidence in physical activity among patients with cardiac physiologic pacing. However, the clinical significance of this improvement should be interpreted cautiously, and future studies should determine whether the observed changes exceed the minimal clinically important difference in this population.

Significant improvement was also found in flexibility outcomes. Upper limb flexibility measured using the Back Scratch Test improved from −20.22 ± 4.80 cm to −19.09 ± 5.14 cm (t = 3.373, p = 0.0013), whereas lower limb flexibility assessed using the Sit-and-Reach Test improved from −21.37 ± 4.67 cm to −18.32 ± 9.30 cm (t = 2.966, p = 0.0043). Restriction of shoulder and trunk mobility is commonly encountered following pacemaker implantation because patients often avoid movement due to discomfort, fear of lead displacement, or prolonged activity limitation [11, 12]. The stretching and mobility components of the exercise program likely contributed to increased tissue extensibility, improved joint mobility, and restoration of normal movement patterns [19, 20]. These improvements may reduce the effort required for everyday tasks and encourage greater confidence in physical activity.

The present findings are consistent with previous studies demonstrating that supervised exercise training can be safely implemented following permanent pacemaker implantation [23, 24]. Defaye et al. reported that contemporary pacemaker management, combined with appropriate clinical follow-up, supports safe participation in physical activity without increasing device-related complications. Similarly, Brubaker and Kitzman highlighted that appropriately prescribed exercise improves chronotropic response, functional capacity, and cardiovascular performance while maintaining patient safety. The absence of major adverse events, lead-related complications, or device malfunctions in the present study further supports these observations and indicates that a structured aerobic and flexibility exercise program can be safely incorporated into the rehabilitation of patients with cardiac physiologic pacing. The observed findings therefore agree with the existing literature supporting supervised exercise as a safe and feasible component of post-pacemaker rehabilitation [24].

An additional advantage of combining physiologic pacing with exercise rehabilitation is the potential to optimize both central and peripheral determinants of physical performance. Physiologic pacing preserves a more natural ventricular activation sequence and minimizes the detrimental effects associated with traditional right ventricular pacing, while exercise training improves muscular, vascular, and metabolic function [5, 8]. These combined effects may help to inhibit progressive functional decline and maintain independence, particularly in older adults who are at increased risk of frailty and age-related muscle loss.

Certain limitations should be considered when interpreting the results. The absence of a control group limits the ability to attribute all observed improvements exclusively to the intervention. Furthermore, the use of convenience sampling may have introduced selection bias and may limit the generalizability of the findings to the broader population of patients with cardiac physiologic pacing. In addition, the relatively small sample size may have limited the statistical power of the study and reduced the generalizability of the findings. Larger multicenter studies are warranted to validate these results. The relatively short duration of follow-up does not permit evaluation of the long-term maintenance of the observed benefits. Objective physiologic measures such as cardiopulmonary exercise testing, peak oxygen uptake (VO₂ peak), and echocardiographic assessment of cardiac performance were not included and could have provided additional insight into the mechanisms underlying the observed improvements. Additionally, detailed device-related characteristics, including pacing burden, device manufacturer, pacing mode, and programming parameters, were not collected and therefore could not be analyzed. Future studies should incorporate these variables to better understand their potential influence on functional outcomes following cardiac physiologic pacing.

Future studies should employ randomized controlled trial designs with larger sample sizes and longer follow-up periods to strengthen the evidence base and confirm the long-term effectiveness of structured exercise programs in this population. Investigation of factors such as pacing burden, pacing mode, device programming characteristics, and associated comorbidities may further clarify their influence on rehabilitation outcomes. Overall, the present findings provide preliminary evidence that a structured program combining aerobic conditioning and flexibility exercises may be a safe and feasible rehabilitation strategy for improving functional capacity and mobility in patients with cardiac physiologic pacing. Further randomized controlled trials are required to confirm these findings.

## Conclusions

The findings of this study indicate that a 12-week structured exercise program incorporating aerobic and flexibility training resulted in significant improvements in functional exercise capacity, shoulder flexibility, and lower limb flexibility among patients with cardiac physiologic pacing pacemakers. Improvements in 6MWT performance, Back Scratch Test scores, and Sit-and-Reach Test scores demonstrate the positive impact of the intervention on functional exercise capacity and musculoskeletal mobility.

The absence of major adverse events throughout the intervention period further supports the feasibility and safety of supervised exercise participation in this population. These findings provide preliminary evidence that structured exercise training may improve physical functioning and mobility following cardiac physiologic pacing. However, given the quasi-experimental single-group study design, the absence of a control group, and the relatively small sample size, these findings should be interpreted with caution. Larger randomized controlled trials with longer follow-up are required to confirm these findings, establish clinical effectiveness, and support routine clinical implementation of exercise-based rehabilitation in this population.
